# HSP60-Derived Peptide as an LPS/TLR4 Modulator: An *in silico* Approach

**DOI:** 10.3389/fcvm.2022.731376

**Published:** 2022-04-01

**Authors:** Rafael Gustavo Vila-Casahonda, Jorge Lozano-Aponte, Carlos Enrique Guerrero-Beltrán

**Affiliations:** ^1^Tecnologico de Monterrey, Medicina Cardiovascular y Metabolómica, Escuela de Medicina y Ciencias de la Salud, Tecnologico de Monterrey, Monterrey, Mexico; ^2^Tecnologico de Monterrey, Escuela de Ingeniería y Ciencias, Tecnologico de Monterrey, Puebla, Mexico

**Keywords:** heat shock protein 60 (HSP60), TLR4/MD2 complex, cardiovascular system, therapy, cardiovascular disease, molecular dynamics, computational modeling

## Abstract

As a part of innate immunity mechanisms, the Toll-like receptor (TLR) signaling pathway serves as one of the mainstay lines of defense against pathogenic microorganisms and cell dysfunction. Nevertheless, TLR overactivation induces a systemic proinflammatory environment compromising organ function or causing the patient’s death. TLRs modulators, specially those focused for TLR4, remain a promising approach for inflammatory diseases treatment, being peptide-based therapy a trendy approach. Heat shock protein 60 (HSP60) not only plays a pivotal role in the development of several maladies with strong inflammatory components but also HSP60 peptides possess anti-inflammatory properties in TLR4-mediated diseases, such as diabetes, arthritis, and atherosclerosis. The experimental treatment using HSP60 peptides has proven to be protective in preclinical models of the heart by hampering inflammation and modulating the activity of immune cells. Nonetheless, the effect that these peptides may exert directly on cells that express TLR and its role to inhibit overactivation remain elusive. The aim of this study is to evaluate by molecular docking, a 15 amino acid long-HSP60 peptide (Peptide-2) in the lipopolysaccharide (LPS) binding site of TLR4/MD2, finding most Peptide-2 resulting conformations posed into the hydrophobic pocket of MD2. This observation is supported by binding energy obtained for the control antagonist Eritoran, close to those of Peptide-2. This last does not undergo drastic structural changes, moving into a delimited space, and maintaining the same orientation during molecular dynamics simulation. Based on the two computational techniques applied, interaction patterns were defined for Peptide-2. With these results, it is plausible to propose a peptidic approach for TLR4 modulation as a new innovative therapy to the treatment of TLR4-related cardiovascular diseases.

## Introduction

Toll-like receptors (TLRs) are a type I transmembrane glycoprotein family responsible for the sensing of pathogen-associated molecular patterns (PAMPs), damage-associated molecular patterns (DAMPs), and certain endogenous ligands. They play a key role in the immune and non-immune system activation, functioning as an interface between the adaptative and innate immune system (1, 2). Upon ligand recognition, an intracellular signaling cascade is activated, which results in the expression of several proinflammatory cytokines, representing a crucial defense mechanism and contributing to survival and cell homeostasis, thereby limiting the impact caused on the host (3). Despite its protective nature, an exacerbated response is tightly associated with acute organ dysfunction and a long-term cardiovascular dysfunction, cancer, sepsis, neurological diseases, and diabetes, to mention some; therefore, there is an interest for further understanding and developing TLR modulating therapies (4, 5).

Among the TLR family, the TLR4 is abundantly expressed on innate immune cells and in almost all somatic cells and tissues (6); thus, the TLR4 becomes the main target of interest for the development of regulatory therapies. The TLR4 possesses the ability to detect miniscule amounts of circulating ligands with a different nature, like DAMPS. It can also detect endogenous ligands (alarmins), such as high-mobility group box 1 protein or heat shock proteins (HSPs), and PAMPs, such as liposaccharides (LPSs). Consequently, TLR4 activation mechanisms remain elusive and modulatory approaches have focused mainly on LPS-derived scaffolds, based on the defined binding site with TLR4 ectodomain and MD2 protein (7, 8). Several LPS-derived antagonists, e.g., Eritoran, have been developed to dampen TLR4 activation. Nevertheless, regardless of their success into murine model for impeding TLR4/MD2 dimerization process by competing for MD2 binding pocket, the clinical phase III protocols have failed to reduce inflammation and to improve the survival of patients (9–11). The development of novel molecules that bear no structural similarity to LPS, such as TAK-242 and Neoseptin-3, proposed a novel approach for TLR4 modulation, because these are now peptides and small molecules are a trendy approach for this purpose due to its ease of synthesis and structural versatility (12–14). The outlook in the near future is not promising, with an expected growth in TLR4 dysregulation aggravated diseases (as previously mentioned), the rise in immunocompromised patients among the rapidly expanding elderly population, the increase in invasive surgical procedures, and the emerging antibiotic resistance (15, 16). Regarding such an alarming scenario, the need for immunoregulatory drugs targeting TLR4 is undeniable.

Heat shock protein 60 is a vastly conserved protein in the evolution, present in both prokaryotic and eukaryotic cells, with a preponderant role into cell homeostasis. HSP60 has been reported to be involved in cellular survival, proliferation, and immunization upon TLR4 recognition and activation (17). Upon cellular surface translocation, in exosomes or extracellularly, HSP60 has been associated with proinflammatory effects and apoptosis, when recognized by non-immune (e.g., cardiomyocytes, endothelium) and immune (e.g., macrophages, dendritic) cells (17). Advances in the HSP60 immune system network suggest that it may also help alleviate the inflammatory environment upon TLR4 recognition in a dose-dependent way, as peptides from HSP60 have been reported to induce an anti-inflammatory effect (18). Promising results have been reported in the application of HSP60 peptides in atherosclerotic plaque reduction, juvenile idiopathic arthritis, diabetes, and several autoimmune and proinflammatory diseases (19–21).

Due the promising preliminary results on HSP60 and it’s derived peptides for pattern recognition receptors (PRR), and specially, TLR4 regulatory therapies, further understanding of the interactions governing the TLR4 activation or inhibition exerted by the ligand is needed to fully exploit this regulatory approach. In this regard, this study aimed to dock a 15-residue-long peptide derived from HSP60 (Peptide-2) into the LPS binding site of TLR4-MD2 by automatic docking and molecular dynamics (MD) to explore the Peptide-2 capacity as a TLR4 antagonist.

## Materials and Methods

### Multiple Sequence Alignment

To identify the degree of similarity between the TLR4/MD2 complex receptor of humans and mice, a multiple sequence alignment was performed with BLAST, using BLOSUM62 as the scoring matrix (22, 23). The amino acid sequences from human TLR4 (hTLR4), MD2 (hMD2), mice TLR4 (mTLR4), and MD2 (mMD2) were obtained from UniProt (24) with the accession numbers O00206, Q9Y6Y9, Q9QUK6, and Q9JHF9, respectively.

### Molecular Modeling

Three dimensional structures of TLR4 and MD2 proteins were obtained from the Protein Data Bank. As an initial input, human TLR4/MD2/LPS (PDB ID:3FXI) and murine TLR4/MD2/LPS (PDB ID: 3VQ2) were obtained and visually inspected with PyMOL (version 2.4.1) (25).

The peptide-2 structure was obtained based on the HSP60-derived peptide sequence from *Mycobacterium bovis* with the aid of the PEP FOLD 3 (26, 27) peptide structure prediction tool. In order to improve and refine the predicted 3D model of the peptide, the output model of the PEP FOLD 3 server was further processed using the GalaxyRefine 2 server (28). Finally, the refined structure was validated using the Ramachandran plot analysis with MOLprobity (29).

### Molecular Docking

To perform molecular docking analysis, TLR4 and MD2 protein 3D structures were obtained from the Protein Data Bank as previously stated, later removing the waters and redundant ligands from the human and murine TLR4/MD2 complex and using the computational efficient Autodock Vina 1.1.2 docking software for the analysis (30). The following were the docking conditions: for the human TLR4/MD2 (hTLR4/MD2) complex, the grid box spacing was set to 1 Å; box grid point dimensions were 33, 40.5, and 35.25 Å (x,y,z); the box center coordinates were 25.692, −5.342, and 14.883 (x,y,z), equidistant to residues Arg90 (MD-2), Lys122 (MD-2), and Arg264 (TLR4) (31). In the case of murine TLR4/MD2, the grid box spacing was set to 1 Å; the box grid point dimensions were 33, 40.5, and 35.25 Å (x,y,z); the box center coordinates were −27.261, −15.196, and 21.148 (x,y,z), in accordance to literature (32, 33). In both cases, the receptor was kept rigid and all rotatable bonds in ligand remained free, the polar hydrogen atoms and Gasteiger charges were assigned. For validation of the docking method, the agonistic LPS extracted from crystallographic structures was re-docked into human and murine TLR4/MD2 complex with the previously stated conditions and the root mean square deviation (RMSD) was obtained.

Due to the lack of complete TLR4/MD2/Eritoran crystallographic structures, the hybrid human central TLR4/MD2/Eritoran (PDB ID = 2Z65) was used as reference in the human TLR4/MD2/Eritoran docking results. The resulting conformations were analyzed with the PyMOL software. Furthermore, the possible interactions were identified with the ShowContacts plugin. The electrostatic potential of the best docked positions of Peptide-2 and the receptor was processed with the adaptive Poisson-Boltzmann Solver (APBS) electrostatics plugin in the software PyMOL (version 2.4.1), and the results are shown in a red-blue scale for negative and positive values, respectively.

### Molecular Modeling for Molecular Dynamics Simulations

The murine TLR4/MD2 monomer coordinates were taken from the 3VQD pdb file (34). The peptide-2 output conformation from Vina was posed at the binding site of TLR4/MD2 X-ray complex in monomeric form employing the SPDBV software (35).

### Molecular Dynamics Simulations

For this study, all the MD simulations were conducted with the OPLS AA/M force field with Gromacs 2020.4 (36–40). The force field includes optimized torsion parameters for proteins and peptides, offering improvements over previous versions of the OPLS AA. These parameters were evaluated in MD simulations with proteins and the results showed a better relationship with experimental data and high-level quantum chemical methods (41).

Peptide/protein topologies and structure coordinates files were generated with the Gromacs 2020.4 “gmx” commands (36–40). The murine TLR4/MD2 monomeric complex with Peptide-2 at the LPS binding site (denoted as TLR4/MD2/Peptide-2) and without Peptide-2 (denoted as TLR4/MD2) were each simulated in the center of a dodecahedral box. Dodecahedral boxes have the advantage of reducing the simulation time by including fewer water molecules in the simulation cell; nonetheless, they have the disadvantage of generating periodic boundary effects. This is generally evident in some simulation time steps as outlier values in plots where distance (both inter- and intramolecular), RMSD, and radius of gyration as a function of time are measured. The simulation box was filled with the extended simple point charge model for water (SPC/E), and the protein/peptide charges were neutralized with counter ions. Prior to MD production, each molecular construct was subjected to steepest descent energy minimization. Afterward, the same were subjected to two equilibration steps in canonical (NVT) and isobaric-isothermal (NPT) assemblies (both with modified Berendsen thermostat, V-rescale, and NPT with Parrinello-Rahman pressure coupling), both at 300 K for 0.1 ns. A MD production was performed in an NPT assembly under Verlet scheme and Parrinello-Rahman barostat at 310 K for 100 ns (50,000,000 steps, dt = 0.002 ps). MD trajectories, interatomic distances, and RMSD calculations were analyzed and obtained with VMD (42); the plots were generated with the Grace Software (43).

## Results

### Possible Interaction Model Between Peptide-2 and Murine TLR4/MD2 Complex by Molecular Docking

Previous *in silico* studies for the rational design of TLR4 modulators consider the LPS binding site as the canonical binding site; nonetheless, as most ligands are derived from LPS structure, it was imperative to perform molecular docking to confirm the ability of Peptide-2 to fit in the therapeutic target due to its different nature (7). Validation of the docking method showed an excellent performance to reproduce the reported binding affinities (32) and crystallographic structures in the murine TLR4/MD2 monomeric (mTLR4/MD2) and dimeric (mTLR4/MD2)^2^ complex with LPS, producing a RMSD of 0.002 and 0.001 Å, respectively (Table 1). Multiple sequence alignment (Supplementary Figure 1) shows a great identity between human TLR4 and murine TLR4 with 68% of identities and 79% of positive matches; in the case of human and murine MD2, the results show 64% of identities and 80% of positive matches, surpassing in both cases the 30% threshold to identify the homology between proteins (44). In the lack of complete TLR4/MD2/Eritoran complex crystallographic data, the results obtained for docked human TLR4/MD2/Eritoran were used as reference for murine TLR4/MD2/Eritoran. With this in mind, and the focus on murine analysis, Eritoran and peptide-2 showed similar binding affinities. These results suggest that Peptide-2’s interaction with the monomeric and dimeric complexes (mTLR4/MD2) and (mTLR4/MD2)2 is energetically plausible.

**TABLE 1:** Theoretical free binding energy for the docking calculations of ligands with the murine TLR4/MD2 complex (PDB ID = 3VQ2) obtained with AutoDock Vina 1.1.2 and theoretical inhibition constant (*K*_*i*_) obtained with the method from Wermuth (79).

	TLR4/MD2			(TLR4/MD2)^2^		
Ligand	Binding Energy (Kcal/mol)	RMSD (Å)	Inhibitory Constant (*K*_*i*_)	Binding Energy (Kcal/mol)	Inhibitory Constant (*K*_*i*_)	RMSD (Å)
LPS	−27.667 ± 0.808	0.002	56.3 μM^#^	−31.033 ± 1.0214	56.3 μM^#^	0.001
Eritoran	−8.73 ± 0.302	*	441.019 ± 202.978 nM	−10.24 ± 0.184	32.174 ± 8.855 nM	*
Peptide-2	−6.58 ± 0.132	*	15.241 ± 2.868 μM	−13.115 ± 0.213	0.255 ± 0.088 nM	*

*States that there is no crystallographic data reported for this ligand; ^#^taken from Dixit (80).

**FIGURE 1:**
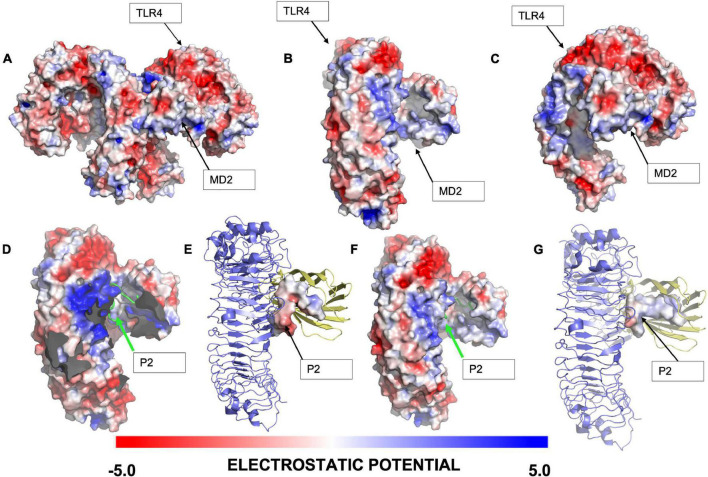
Electrostatic analysis of the interaction of the highest ranked docking conformation of Peptide-2 with murine TLR4/MD2 complex (PDB ID = 3VQ2) obtained with Autodock Vina 1.1.2 and generated with PyMOL 2.4.1. **(A)** General view of (mTLR4/MD2)^2^ electrostatic potential. **(B)** Frontal view of (mTLR4/MD2) electrostatic potential. **(C)** Lateral view of (mTLR4/MD2) electrostatic potential. **(D)** Best docked conformation for Peptide-2 with (mTLR4/MD2)^2^, with complementary TLR4/MD2 not shown for visualization purposes. **(E)** Electrostatic potential of best docked peptide conformation of Peptide-2 with (mTLR4/MD2)^2^. **(F)** Best docked peptide conformation for Peptide-2 with (mTLR4/MD2). **(G)** Electrostatic potential of best docked conformation of Peptide-2 with (mTLR4/MD2). The electrostatic potential is shown in a red (–5) to blue (+ 5) scale obtained with the APBS electrostatics plugin in PyMOL (v.2.4.1).

A first approach was to evaluate the electrostatic potential of (mTLR4/MD2) and (mTLR4/MD2)^2^, respectively, and in the presence of Peptide-2. It has been established that negative surface potential TLR4 interacts with positive surface potential core receptor MD2 by means of charge complementarity (45) (Figures 1A–C). The resulting docking peptide conformations for Peptide-2 with (mTLR4/MD2) and (mTLR4/MD2)^2^ showed a similar potential and conformation; in both cases, the first 10 residues of Peptide-2, identified as N-terminal, expressed a positive electrostatic potential, compatible with the negative charge present at the MD2 entrance rim. On the other hand, terminal 5 residues of Peptide-2, identified as C-terminal, showed a negative electrostatic potential and was oriented toward a positively electrostatic potential spot in TLR4 (Figures 1D–G). These results suggest that Peptide-2 displays fit compatibility with the LPS binding site in terms of electrostatic potentials.

**FIGURE 2:**
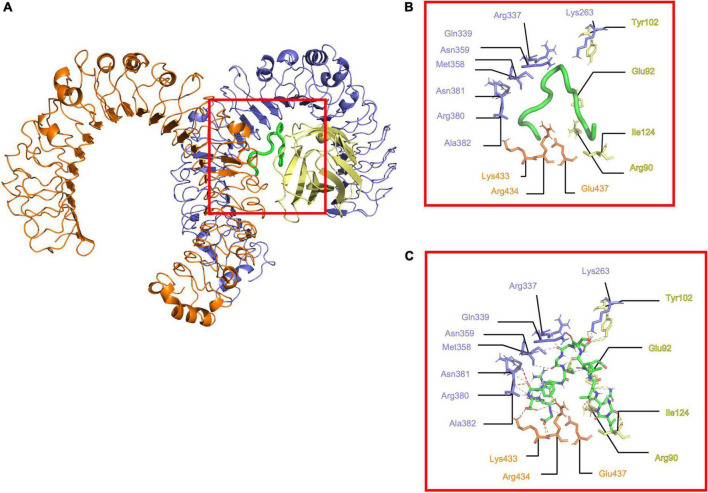
Determined interactions between Peptide-2 and (mTLR4/MD2)^2^. **(A)** General view of Peptide-2 (red square) docked into (mTLR4/MD2)^2^ (TLR4 is shown in purple, MD2 in yellow, and TLR4* in orange). **(B)** Detailed residues interacting with Peptide-2 colored in a segment belonging manner. **(C)** Detailed interactions occurring between residues and Peptide-2 (showed in a licorice representation with carbon atoms in green, oxygen atoms in red, hydrogen atoms in white, and nitrogen atoms in blue), hydrogen bonds are shown as a yellow dashed line, electronic clashes in a red dashed line, and electrostatic interactions in a magenta dashed line. All images were obtained with PyMOL and interactions identified with the ShowContacts plugin.

The docking results were processed to identify the residues involved in interaction. The (TLR4/MD2)^2^ showed 15 electrostatic interactions with different segments of the receptor (Figures 2A–C). Residues 1-8 from the N-terminal of Peptide-2 are embedded into the MD2 pocket, with hydrophobic residues Phe121, Ile124, and Phe126 around; on the other hand, residues 9–15 from the C-terminal of Peptide-2 exhibit a proximity to TLR4 and TLR4*. A detailed description of interacting residues, distance, and atoms involved can be found in Table 2.

**TABLE 2:** Interacting residues and distance of the best obtained docking peptide conformation with Autodock Vina 1.1.2.

(TLR4/MD2) COMPLEX	Peptide 2	Distance (Å)	(TLR4/MD2)^2^ COMPLEX	Peptide 2	Distance (Å)
Arg90/A/NH1	Arg11/NH2	3.7	Arg90/A/1HH1	Gln3/O	2.6
Lys91/A/O	Lys14/NZ	2.9	Arg90/A/1HH2	Gln3/O	2.4
Lys91/A/O	Lys14/HZ3	2	Arg90/A/NH1	Gln3/O	3.2
Glu92/A/OE2	Gly15/O2	3.5	Arg90/A/NH2	Gln3/O	3.1
Glu92/A/OE2	Arg11/NE	4	Arg90/A/NH2	Gln3/N	3.4
Glu92/A/OE1	Arg11/HE	1.9	Glu92/A/OE1	Gln3/NE2	3.3
Glu92/A/OE1	Arg11/NE	2.8	Tyr102/A/HH	Glu8/OE2	2.7
Glu92/A/OE1	Arg11/1HH2	2.4	Tyr102/A/OH	Glu8/OE2	3.5
Glu92/A/OE1	Arg11/NH2	3.2	Ile124/A/HN	Gly1/O	2.4
Glu92/A/OE1	Glu8/OE 2	3.6	Ile124/A/N	Gly1/O	3.2
Val93/A/HN	Gly15/O2	2.4	Lys263/B/HZ1	Thr7/OG1	2.4
Val93/A/N	Gly15/O2	3.2	Lys263/B/NZ	Thr7/OG1	2.8
Val93/A/O	Gly15/O2	3.1	Arg337/B/O	Glu8/O	3.7
Val93/A/O	Gly15/O1	3.3	Gln339/B/OE1	Thr7/O	3.5
Val93/A/O	Met10/O	3.6	Met358/B/O	Glu8/O	3.7
His96/A/NE2	Gly15/O1	3.6	Asn359/B/O	Gly9/O	4
His96/A/HE2	Gly15/O1	3	Arg380/B/O	Phe12/O	3
Tyr102/A/OH	Met10/N	3.2	Asn381/B/N	Phe12/O	3.6
Asn359/B/O	Asp13/OD1	3.8	Ala382/B/HN	Phe12/O	2.5
Gly361/B	Asp13/OD1	3.7	Ala382/B/N	Phe12/O	3.1
Arg380/B/O	Asp13/OD2	3.3	Ala382/B/N	Asp13/N	3.9
Asn381/B/N	Asp13/OD2	3.9	Lys433/C/HZ2	Asp13/OD2	3
Ala382/B/HN	Asp13/OD2	2.8	Lys433/C/NZ	Asp13/OD2	3.8
Ala 382/B/N	Asp13/OD2	3.1	Arg434/C/HN	Gly15/O2	2.5
			Arg434/C/N	Gly15/O2	3.1
			Arg434/C/NH1	Arg11/O	2.8
			Arg 434/C/NH1	Asp 13/OD1	3.9
			Arg 434/C/1HH1	Arg 11/O	2.3
			Arg 434/C/1HH1	Asp 13/OD1	3.3
			Glu 437/C/OE1	Gly 15/N	3.9

*Chain A represents MD-2 subunit, chain B the TLR-4 and chain C represents TLR-4 complementary of the murine (TLR4/MD2) (PDB ID = 3VQ2).*

**FIGURE 3:**
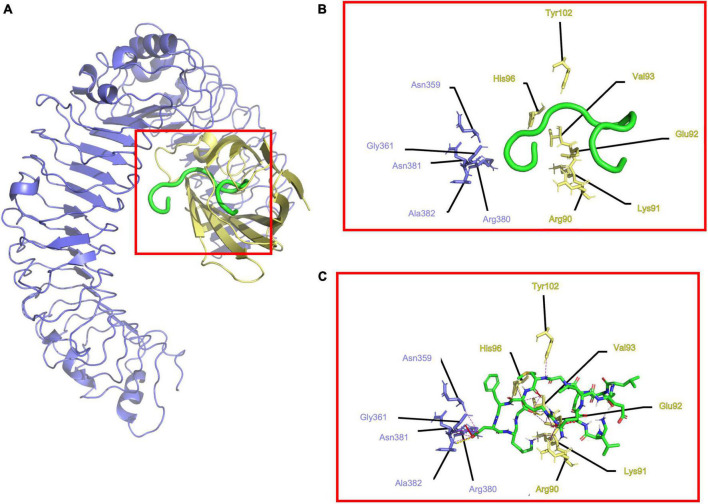
Determined interactions between Peptide-2 and mTLR4/MD2. **(A)** General view of Peptide-2 (red) docked into mTLR4/MD2 (TLR4 is shown in purple and MD2 in yellow). **(B)** Detailed residues interacting with Peptide-2 colored in a segment belonging manner. **(C)** Detailed interactions occurring between residues and Peptide-2 (showed in a licorice representation with carbon atoms in green, oxygen atoms in red, hydrogen atoms in white, and nitrogen atoms in blue), hydrogen bonds are shown as a yellow dashed line, electronic clashes in a red dashed line, and electrostatic interactions in a magenta dashed line. All images were obtained with PyMOL and interactions identified with the ShowContacts plugin.

In the case of (TLR4/MD2), it showed 11 electrostatic interactions (Figures 3A–C). Hydrophobic residues 1–9 from the C-terminal of Peptide-2 fit into the MD2 hydrophobic cavity, surrounded by hydrophobic Ile80, Val82, Leu87, Pro118, Phe119, Ser120, Phe121, Glu122, Ile124, Gly123, Phe126, Pro127, Tyr131, Cys133, and Ala135, to 3.5 Å. A difference between (TLR4/MD2) and (TLR4/MD2)^2^ can be observed in residues 10–15 from the N-terminal of Peptide-2 being less exposed to the solvent, to the Phe12 side chain from Peptide-2 closer to Arg337, Met358, and Lys360. Further detail of the interactions can be seen in Table 2.

Finally, to compare and determine the important interactions for TLR4 modulation, a per subunit matrix of residues was developed as shown (Supplementary Figure 2). Based upon this, we could see common interactions between the canonical agonist LPS and the antagonist Eritoran with our Peptide-2 model. This result suggests that further analysis should be done to determine the possible Peptide-2 behavior as a TLR4 modulator.

### Molecular Dynamics of Murine TLR4/MD2 Monomeric Complex and Peptide-2

**FIGURE 4:**
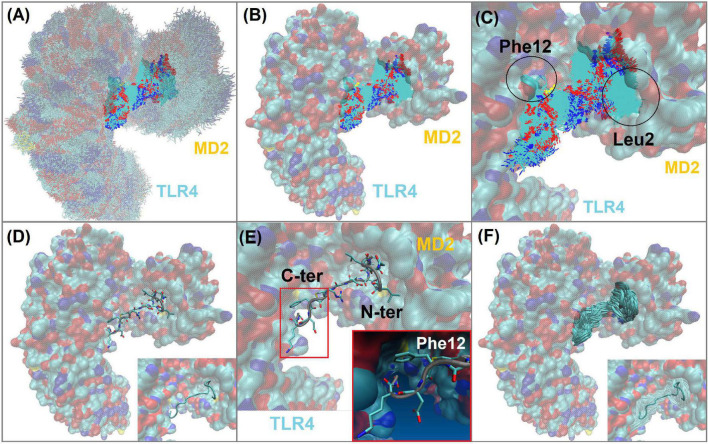
TLR4/MD2/Peptide-2 complex simulated by molecular dynamics at 310 K for 100 ns. **(A)** Molecular dynamics (MD) trajectory superposition of TLR4/MD2/Peptide-2 MD. **(B)** Peptide-2 trajectory superposition on TLR4/MD2 complex (surfaced by atom color). **(C)** Close-up of panel **(B)**, denoting the trajectory of Leu2 and Phe12 residues of Peptide-2 interacting with hydrophobic sites of MD2 and TLR4, respectively. **(D)** Representation of Peptide-2 (stick) at TLR4/MD2 LPS binding site (tube representation below). **(E)** Close-up of panel **(D)** showing Peptide-2 orientation and the small hydrophobic cleft of TLR4 where Phe12 side chain takes place during simulation. **(F)** Peptide-2 trajectory superposition (tube) on TLR4/MD2 complex (surfaced by atom color). Resulting trajectories were aligned with VMD “Trajectory tool” and were tracked every 10 steps over a total of 10,000, giving a total of 1,000 superposed conformations. All molecules are displayed without hydrogen atoms. All images were taken at 60 ns.

Peptide-2 orients hydrophobic residues toward the MD2 cavity where they find an appropriate environment, while polar groups are exposed to the solvent or to the formation of polar interactions (H-bonds) detailed later. Peptide-2 occupies a space that, although not completely defined, is clearly delimited (Figures 4A–F). In this position and orientation, the first ten residues (starting at N-terminal Gly1) are surrounded by MD2 residues, and the rest (finishing at C-terminal Gly15) are closer to TLR4. The first eight residues of Peptide-2 are embedded in the hydrophobic pocket of MD2. This position and the observed interactions do not change drastically with the passage of simulation time.

**FIGURE 5:**
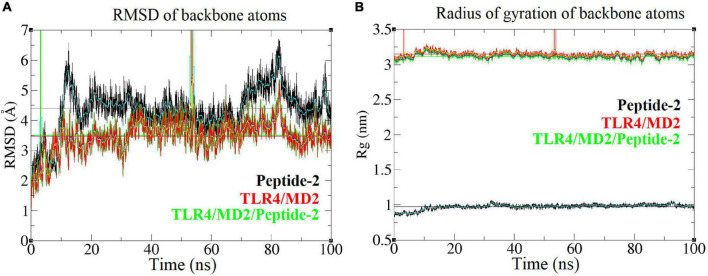
Root mean square deviation and radius of gyration of different backbone molecules of the TLR4/MD2/Peptide-2 complex simulated by MD at 310 K for 100 ns. **(A)** Root mean square deviation or RMSD (nm, Y axis) vs. time (ns, *X* axis) plot, **(B)** Radius of gyration or Rg (nm, *Y* axis) vs. time (ns, *X* axis) plot light blue, navy blue, and yellow lines represent the average value between each simulation step. To calculate and plot the RMSD, the MD resulting conformations were previously aligned using the “Trajectory tool” of VMD package (42). Vertical green and red lines are due periodic boundary effects.

Radius of gyration (Rg) is an approximate measure of the compactness and global shape of a molecular structure; it is roughly calculated considering the mass and position of each atom, with respect to the center of mass of the molecule (46). A linear-like behavior on the Rg vs. time plot representation indicates that the shape of the molecule does not undergo significant changes during the simulation (Figure 5B). With respect to the RMSD a value less than 2.5 Å (0.25 nm) has been arbitrarily taken as a threshold or limit value to determine a low atom deviation or (in other words) that the molecule maintains structural stability. Although there is nothing that fully justifies the aforementioned criterion, it is known that the lower this value, the greater the stability of a molecular structure (Figure 5A) (47).

When plotting the RMSD vs. time of the TLR4/MD2/Peptide-2 complex backbone, it provides an approximate value of 3.5 Å, which although is high, remains stable over the simulation time. These plots show that parts of the receptor suffer periodic boundary effects without significantly affecting these measurements (green and red vertical lines between 0 and 10 ns and between 50 and 60 ns, Figure 5). Peptide-2 experiences a higher structure deviation, reflected in an average RMSD value clearly higher than those observed with TLR4/MD2/Peptide-2 and TLR4/MD2 complexes (Table 3).

**TABLE 3:** Mean values of RMSD and Rg calculated for different molecular structures simulated by molecular dynamics of the TLR4/MD2/Peptide-2 complex at 310 K for 100 ns.

Protein/Peptide	RSMD (Å)	Rg (nm)
Peptide-2	4.408	0.9758
TLR4/MD2	3.471	3.1483
TLR4/MD2/Peptide-2	3.495	3.1114

Comparing the MD simulations of the TLR4/MD2 monomeric complex in the presence and in the absence of Peptide-2, it is observed that in the presence of this, the monomer suffers higher structural deviation and greater form variation; even though it is not notable visually when superimposing the MD trajectories, the results are evident both graphically and numerically (RMSD and Rg plots, Supplementary Figure 3). In other words, the presence of Peptide-2 causes a statistically significant reduction in the structural stability of the receptor complex when simulated by MD (Supplementary Table 1).

### Hydrogen-Bond Interactions Between TLR4/MD2 Monomeric Complex and Peptide-2 Observed in Function of Time by Molecular Dynamics Simulations

**FIGURE 6:**
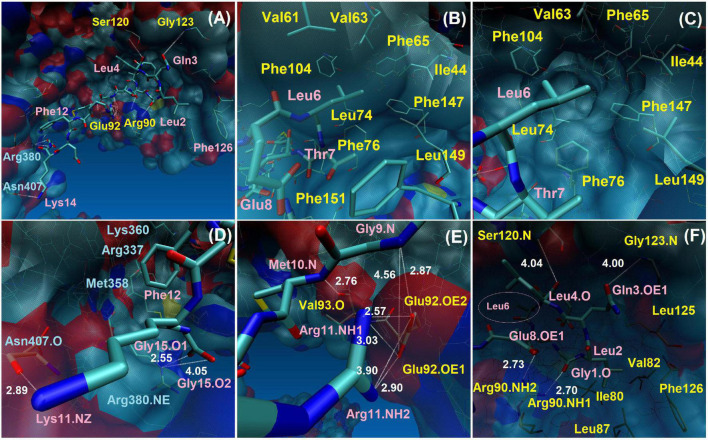
Interactions between TLR4/MD2 monomeric complex and Peptide-2 observed by MD simulations at 310 K for 100 ns. **(A)** Full perspective of Peptide-2 binding mode with TLR4/MD2 complex, only a few residues are included for positional reference, details and close-ups are shown below. **(B,C)** Different perspectives of the MD2 hydrophobic pocket and its residues around Leu6 and contiguous residues of Peptide-2. **(D)** Interactions of TLR4 with Peptide-2 residues. **(E,F)** Interactions of MD2 with Peptide-2 residues (Leu6 lies deep within the MD2 binding site, left side of panel **(F)**, smaller label at pink circle). Blue labels: TLR4 residues. Pink labels: Peptide-2 residues. Yellow labels: MD2 residues. Only H-bond distances were measured (in angstroms or Å, white labels, white dotted lines). Single O or N: backbone residue atoms. NH1, NH2: nitrogen atoms at the end of the arginine side chain. NE: nitrogen epsilon atom of arginine side chain. NZ: nitrogen atom of lysine side chain. O1, O2: glycine 1 oxygen atoms at the C-terminus of Peptide-2. OE1, OE2: oxygen atoms of glutamic acid side chain. All images were taken at 60 ns, at which point most interactions are noticeable. More details in Supplementary Table 2.

Molecular dynamics simulations show two types of well-defined interactions: (i) interactions between residual polar atoms or H-bonds (48), being their distance measured as a function of time (Supplementary Table 2), and (ii) hydrophobic interactions with the MD2 binding site and with localized regions of TLR4 (Figure 6). Three interactions stand out showing a constant distance below 3 Å during practically the entire simulation. The backbone nitrogen (N) atoms of the Peptide-2 residues Met10 and Leu4 form interactions with the oxygen (O) backbone atoms of Val93 and Ser120 of MD2 (Figures 6E,F), respectively, presenting an interaction distance below 3 Å for all or most of the simulation time; thus, these are considered the most stable interactions in terms of distance. The interaction of nitrogen atoms of Glu9 backbone and Arg11 side chain of Peptide-2 (N and NH1, NH2, respectively), with oxygen (OE1, OE2) atoms of Glu92 side chain of MD2, occur alternately due to the rotation of the carboxyl group of Glu92; nevertheless, this allows the distance to the two Peptide-2 residues to remain nearly constant throughout most of the simulation.

**FIGURE 7:**
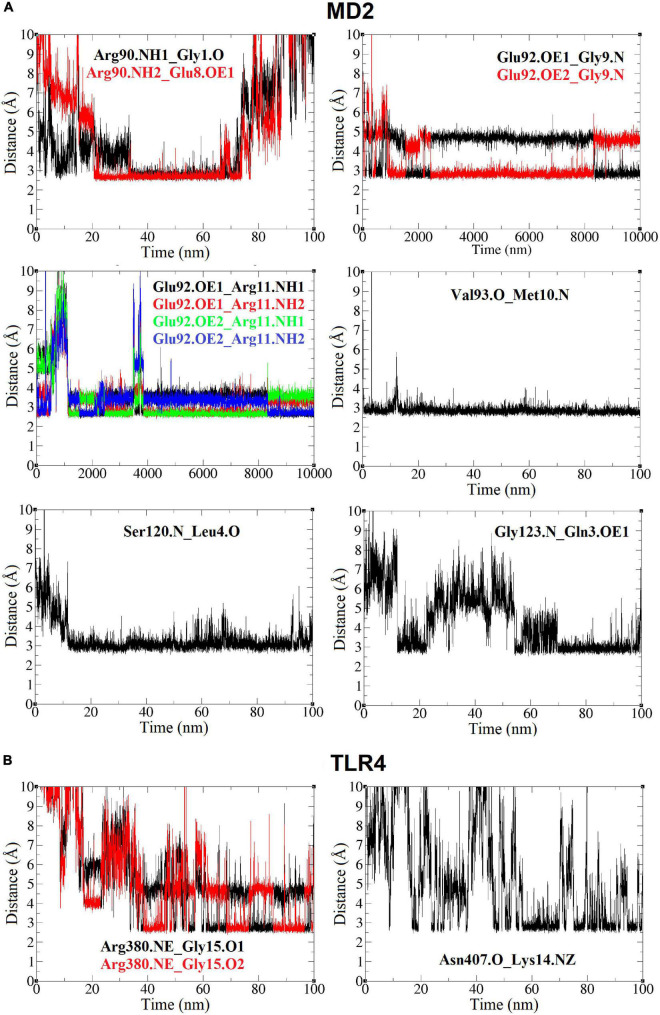
Distance (Å, *Y* axis) vs. time (nm, *X* axis) plots between TLR4/MD2 and Peptide-2 residues, for the TLR4/MD2/Peptide-2 complex simulated by MD at 310 K for 100 ns. **(A)** Plots (yellow box) for MD2 residues. **(B)** Plots (blue box) for TLR4 residues. In all notations, the left residue belongs to the TLR4 or MD2 subunit and the residue at right is from Peptide-2. Single O or N: residue backbone atoms. NH1, NH2: nitrogen atoms at the end of the arginine side chain. NE: nitrogen epsilon atom of arginine side chain. NZ: nitrogen atom of lysine side chain. O1, O2: glycine 1 oxygen atoms at the C-terminus of Peptide-2. OE1, OE2: oxygen atoms of glutamic acid side chain.

Other observed interactions are intermittent and show very high distances at some moments of the simulation. In this way, it includes the interaction of oxygen atoms of Gly1 backbone and Glu8 side chain of Peptide-2 (O and OE1, respectively), with nitrogen (NH1, NH2) atoms of Arg90 side chain of MD2 (Figure 6F), which occur almost simultaneously after 20 ns and is maintained for approximately 50 or 60 ns, mainly with Glu8, time in which the distance is also kept below 3 Å, too. The side chains of both Glu8 (Peptide-2) and Arg90 (MD2) are exposed to the solvent (water), which explains why the interaction is not evident until after 20 ns and breaks after 80 ns. The Gly123 residue is on the MD2 binding site periphery; the interaction of backbone nitrogen atom of this residue with Gln3 side chain oxygen of Peptide-2 occurs between 10 and 20 ns approximately, but it is until after 50 ns that it presents a consistent distance below 3 Å until the end of the simulation (Figure 7A).

Only two TLR4 residues showed interactions with Peptide-2 (Figure 6D). At almost 40 ns of the simulation, it is observed that the nitrogen (epsilon, NE) of Arg380 side chain contacts Peptide-2 C-terminal oxygen (O1, O2) atoms of Gly15, alternately maintaining a distance slightly less than 3 Å (by rotation of carboxyl group) from that moment to the end of the simulation. The interaction of backbone oxygen of Asn407 with Lys14 side chain nitrogen is less defined and goes unnoticed for almost half the simulation time, at which point it becomes noticeable without being entirely constant or consistent in terms of distance (Figure 7B). The Lys14 residue is in contact with water, so it is not surprising that this interaction is the most intermittent among those that have been presented so far; nonetheless, it is a contribution for the interaction with TLR4.

### Hydrophobic Interactions Between TLR4/MD2 Monomeric Complex and Peptide-2 Observed by Molecular Dynamics Simulations

Three hydrophobic residues appear to be decisive in Peptide-2 binding mode with the TLR4/MD2 complex: Leu2, Leu6, and Phe12. Mainly, the interactions of the Leu2 and Leu6 residues at Peptide-2 N-terminal determine its orientation (Figures 4C,E, 6B,C). Both residues are favorably embedded in the hydrophobic pocket of MD2, Leu2 side chain does so in the part furthest from TLR4, close to the Phe126 residue, while Leu6 side chain is immersed in a cavity close to TLR4 (Figures 6C,E).

**FIGURE 8:**
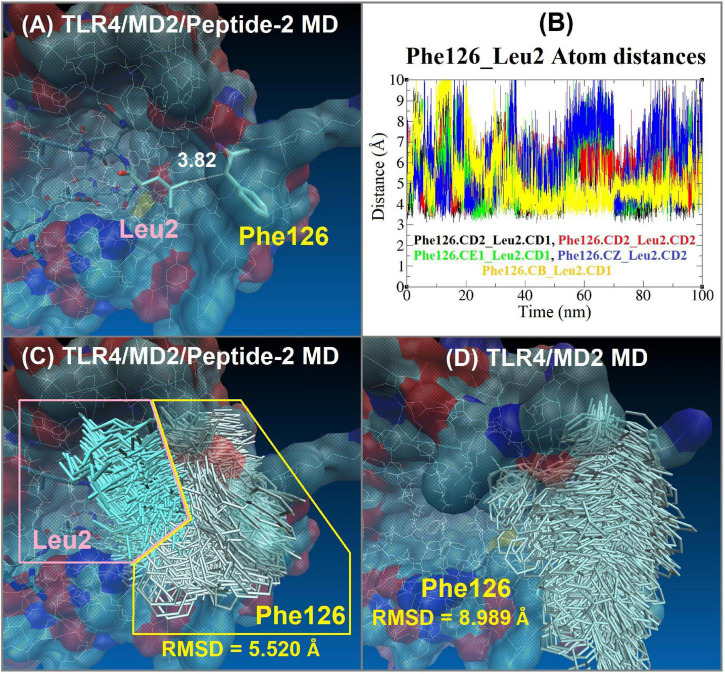
Interaction between Phe126 of MD2 and Leu2 of Peptide-2 observed by MD simulations at 310 K for 100 ns. **(A)** Interaction distance between Peptide-2 Leu2.CD1 and MD2 Phe126.CB, yellow line in plot **(B)** (the most consistent interactions in terms of distance). **(B)** Distance (Å, *Y* axis) vs. time (nm, *X* axis) plot between different carbon atoms of Phe126 and Leu2 residues, merging all the distance vs. time plots allows observing that these residues are at a distance of less than 4 Å during practically the entire simulation. CB: Carbon beta of Phenylalanine residue. CE1: Carbon epsilon 1 of Phenylalanine residue. CD1, CD2: Carbon delta 1 and 2 of Leucine or Phenylalanine residues. CZ: Carbon zeta of Phenylalanine residue. **(C)** Side chain MD-trajectories superposition of Phe126 (silver) and Leu2 (light blue) residues, remaining very close during the simulation time. Plots **(A–C)** correspond to the TLR4/MD2/Peptide-2 MD simulation at 310 K for 100 ns. **(D)** Side chain MD-trajectories superposition of Phe126 (silver) observed with the TLR4/MD2 MD simulation in absence of Peptide-2 at 310 K for 100 ns [panels **(C,D)** trajectories were aligned with VMD “Trajectory tool” and were tracked every 10 steps over a total of 10,000, giving a total of 1,000 superposed conformations]. Average RMSD values of Phe126 side chain atoms are shown at the bottom of panels **(C,D)** (side chain hydrogen atoms were not taken account for these calculations).

In addition to the above, the closeness and interaction of Peptide-2 Leu2 with MD2 Phe126 cause the latter’s side chain to remain in the hydrophobic environment of MD2 (Figure 8), which in the presence of oligosaccharide-type ligands has been associated with a receptor agonist activity (49). However, the scope of this work does not allow an in-depth discussion of the Peptide-2 effect on the TLR4/MD2 receptor complex.

A non-evident interaction is the one established between the Phe12 residue of Peptide-2 and the side chains of Arg337, Met358, and Arg360 of TLR4 (Figures 4C,E, 6D). Appealingly, Arg360 have been recognized as the site for electrostatic or polar interactions with LPS and derivatives (34, 50, 51); yet in this case, the side chains of the mentioned residues form a small hydrophobic cleft in which the phenyl group of Phe12 fits.

## Discussion

TLR4 overactivation is a key contributor for the progression of several pathologies such as heart failure, atherosclerosis, sepsis, arthritis, autoimmune disorders, diabetes, etc., being its main effect to evoke an inflammatory response that alters multiple organs and cell populations, where the PRRs are present (52). TLR4 modulation represents both a challenge and an opportunity as a therapeutic tool, due to its key role on cell homeostasis and diseases, with different inhibitory ligands (such as Eritoran and TAK-242), failing at different stages in clinical trials. Nonetheless, novel approaches as the use of naturally derived molecules such as berberine or small molecules such as Neoseptin-3 suggest that other ligands may achieve what has been utterly difficult to attain, making plausible the development of treatments for diverse inflammatory-related diseases (53–55). Cardiovascular diseases are the leading cause of mortality worldwide, where heart failure has its special place within this group of maladies, serving as common ground between all of them, and representing the terminal stage of these diseases. The sum of noxious events occurring in TLR4 imparity at different cell populations represent the majority of the causes associated to the related dysfunction; however, in the cardiovascular system, the involvement of other cells expressing TLR4, e.g., endothelium, cardiomyocytes, as well as other anatomical structures important for cardiac dynamics, plays a role on the pathophysiology of cardiovascular diseases (45, 56).

There have been multiple efforts for the development of TLR4 modulators to decimate its inflammatory effects, but all the proposed molecules have failed at different stages of clinical trials, leaving us with no FDA-approved treatment option for some TLR4-dysregulated diseases (9–11). Among endogenous molecules, several represent a high conservational degree throughout evolution, thus being present in prokaryotic and eukaryotic organisms. An example of this is HSP60, which is found in both bacteria and multicellular organisms, including humans. For the case of the latter species, exposure to HSP60 may occur either as a PAMP or a DAMP, depending on the source of the protein (57). Owing to the high homology degree between the bacterial HSP60 and its human counterpart, when the latter is released into the extracellular space it may be detected by TLR4, a phenomenon known as molecular mimicry, whereby an endogenous element is identified as an antigen by the cells of the immune system, as a result of its strong resemblance to its pathogenic cognates (58). Advances in HSP60 immune system network suggest that the peptides derived from this protein may also be helping to alleviate the inflammatory TLR4-related effects, as peptides derived from this protein have reported to have anti-inflammatory effects with promising results on autoimmune and cardiovascular diseases, which are pathologies where TLR4 also represents a key role (18, 19, 59, 60). Although still at very early stages, TLR4 modulators remain a promising approach, being peptide-based treatments a trendy approach for their benefits on other highly specific therapies, such as antibodies therapies, but with a shorter half-life, lower production cost, and increased product reproducibility, showing interesting results in the heart and vessels (61, 62).

With the increasing technological advances, computer-aided drug design techniques have made possible the design of decoy peptides capable of disrupting TLR4 signaling at different levels (9, 63). In this regard, this study focuses on understanding the interactions at molecular level between the HSP60-derived Peptide-2 and the murine TLR4/MD2 receptor complex, through computational simulation methods, taking as reference the canonical agonist and antagonist molecules, LPS and Eritoran, respectively.

Another study shows that the variability in the length, shape, and amino acid composition of peptide ligands has not allowed establishing a convincing relationship between the molecular aspects and the regulatory effects of the TLR4 observed (64). In turn, the binding mode seems very particular to the peptide analyzed, in contrast to the anchor points consistently reported for LPS-type ligands (33, 34, 54, 65). In the absence of crystallographic information, computational approaches can provide theoretical information at the molecular level that helps to decipher the functional interaction between the ligand and the receptor (66, 67).

In this study, molecular docking showed a plausible Peptide-2 conformation in the murine TLR4/MD2 complex, according to previous reports (Supplementary Figure 2). As can be seen (Figure 1), electrostatic interactions play a governing role in the activation of the TLR4/MD2, conferring complementary charges between the negatively charged TLR4 and the positively charged MD2 co-receptor. Peptide-2-predicted peptide conformations showed similarities between the (mTLR4/MD2) and (mTLR4/MD2)^2^ docking results, showing a positive potential at the first ten residues (starting at N-terminal Gly1), closer to the negative entrance of MD2 receptor (Figures 1D–G). The rest of the Peptide-2 residues (C-terminal Gly15) with overall negative charge are oriented toward a positively charged spot in TLR4, showing electrostatic compatibility among conformations of Peptide-2 with the receptor (Figures 1D–G). Electrostatic compatibility is crucial in the interactions between phosphate groups of LPS and Eritoran, playing an anchoring role in the ligand coupling (51, 54), and in this regard, mimicking these interactions would increase the affinity of docking peptide conformations.

Experimental inhibition constants (*K*_*i*_, Table 1) for Peptide-2 resemble that of experimental Eritoran more than LPS, which, in terms of binding energy, can only be seen as a very similar affinity; nonetheless, these values are not comparable due to the evident chemical structure differences. Furthermore, possible mathematical overestimation of intermolecular interaction forces could happen due to the summative nature of the Autodock Vina scoring function (30) (Supplementary Figure 4).

Peptide-2’s interaction inspection with (mTLR4/MD2)^2^ showed fifteen electrostatic interactions (Figures 2A–C) in comparison to the eleven found with (mTLR4/MD2) (Figures 3A–C), reflected in their binding energies. In the case of (mTLR4/MD2)^2^, it is of peculiar interest that residues 1–8 of the Peptide-2 N-terminal are embedded into the MD2 pocket, with hydrophobic residues Phe126, Ile124, and Phe121 around this Leucine-rich section of Peptide-2, evidencing the weight of the hydrophobic interactions for any ligands to couple into the MD2 hydrophobic pocket, a fact that has been reported in other TLR4/MD2 ligands (1, 7, 53, 68, 69).

Residues such as Arg90, Tyr102, and Glu122 of MD2 subunit have been reported to act as anchor sites for other ligands such as LPS, stabilizing the adopted conformation (32, 33). Moreover, electrostatic interactions with Lys263 and Lys360 of TLR4 and Arg434 and Glu437 of TLR4* subunits of (mTLR4/MD2)^2^ complex have been reported for LPS (32, 33). Residues 9–15 of Peptide-2 toward C-terminal, exhibit proximity to TLR4 and TLR4*, compatible with the opposite charge of this region (Figures 1D,E), as well as the presence of Glu8 and Asp13 in Peptide-2, interacting with the Arginine-rich environment (Table 2). TLR4 residues Met358, Ala382, Asn359, Asn381, and Gln339 provide a hydrophobic environment, making possible the coupling of Peptide-2 by interaction with the hydrophobic Thr7, Gly9, Met10, Phe12, and Gly15 at C-terminal.

A smaller number of Peptide-2 interactions with (mTLR4-MD2) compared to (mTLR4/MD2)^2^ were to be expected due to the lack of complementary TLR4*. A point in common between analyses is that residues 1–9 at Peptide-2, facing toward N-terminal, fit into the MD2 hydrophobic pocket, surrounded by Val82, Tyr131, Leu87, Ile80, Pro118, Phe119, Ser120, Phe121, Glu122, Ile124, Gly123, Phe126, Pro127, Tyr131, Ala 135, and Cys133, to 3.5 Å around Leu2 from Peptide-2, newly stating the relevance of hydrophobic interactions, with the only difference of Peptide-2 being more deeply buried in the MD2 cavity. It is interesting that despite maintaining a similar conformation in both cases, when docked into (TLR4/MD2), the N terminal of Peptide-2 shows an interesting change, by flipping the Phe12 side chain from Peptide-2 closer to Arg337, Met358, and Lys360 into a structure that resembles a pocket where hydrophobic interactions occur, giving stability to this conformation.

Residues Arg90, Tyr102, Glu122 from MD2, Lys263, Lys360 from TLR4, and Arg434, Glu437 from TLR4* have been identified as anchoring sites for the LPS-like ligands, in conformations with agonist and antagonist effects (Supplementary Figure 2) (33, 51, 54). Coupled with this, the docking results for Peptide-2 show resemblance to those reported for Eritoran, showing interactions to MD2 residues Arg90, Glu92, Val93, Tyr102, and Ser120, which have also been reported to play a role in ligand anchoring once in contact with MD2 subunit.

However, and despite the coincidences, an agonist or antagonist effect cannot be assumed, since, as mentioned above, the observed effect of Eritoran cannot be extrapolated to Peptide-2. It should be noted that this study constitutes a first description at the molecular level about the binding mode of HSP60 Peptide-2 with the TLR4/MD2 complex. To establish a clear picture of the functional interactions for this type of ligand, it is necessary to analyze other modulator peptides at the molecular level, as well as the evaluation of their biological effect.

Molecular dynamics results support the molecular docking observations for murine monomer. The RMSD and Rg mean values indicate that TLR4/MD2/Peptide-2 does not undergo significant variations in shape and size during simulation, which could be caused by the interaction with Peptide-2. In addition, Peptide-2 showed movement in a delimited space and maintains the same orientation during MD simulation, with the N-terminal to the MD2 and the C-terminal to the TLR4. In our analysis, the MD indicated the presence of electrostatic interactions by means of hydrogen bonding at a constant distance of less than 3 Å during practically the entire simulation between Glu92, Val93, and Ser120 of MD2 with Arg11, Met10, and Leu4 of Peptide-2, respectively. Although molecular docking and MD are different computational tools and approaches, both showed the Val93(MD2)_Met10(Peptide-2) and Glu92(MD2)_Arg11(Peptide-2) interactions. It should be emphasized that the interactions observed by docking and those observed by MD do not necessarily have to coincide, as in molecular docking, coupled peptide conformations depend entirely on the rotatable bonds of the ligand, which will try to accommodate itself in a rigid binding site, whereas in MD, in addition to the energy minimization and equilibration processes, the atom positions change on each simulation step, which may favor some interactions, but can weaken or break others, and the presence of water molecules can also alter or break interactions momentarily or permanently.

Molecular dynamics allows us to observe the closeness between the backbones of Peptide-2 and TLR4/MD2 complex. The interaction of Val93 (MD2) with Met10 (Peptide-2) is close to the Phe12 (Peptide-2) residue, so a repulsive effect of its aromatic side chain with cationic residues of TLR4 could avoid the optimal interaction between Val93 and Met10, but surprisingly, the opposite occurs.

Similar to what was observed with molecular docking, MD highlights the importance of hydrophobic interactions, as the only two observed TLR4 interactions were Arg380(MD2)_Gly15(Peptide-2) and Asn407(MD2)_Lys14(Peptide-2), and have been observed and reported with other previously studied molecules, whether derived from LPS or not (34, 50, 54, 65, 70); nonetheless, they are intermittent (Figure 7B), in this case, due to contact with water. Hydrophobic interactions seem to be critical for a favorable accommodation of Peptide-2 with the TLR4. Leu2 and Leu6 from Peptide-2 are embedded in the hydrophobic environment of MD2, with Leu2 side chain in a hydrophobic cleft close to Phe126 of MD2, and Leu6 in a hydrophobic cavity close to TLR4 (Figures 6C,E). If both residues exchanged their position, the Peptide-2 C-terminal would be oriented toward the solvent and not toward the TLR4 subunit. Since the Peptide-2 C-terminal does not have many hydrophobic residues, an “inverted” or “flipped” binding mode of Peptide-2 within the MD2 cavity seems unlikely. Furthermore, the hydrophobic interaction of Phe12 (Peptide-2) is also observed in the molecular docking (mTLR4/MD2), whose side chain is immersed in a small cleft formed by the Arg337, Met358, and Lys360 side chains of the TLR4 subunit.

By evaluating the interaction distance as a function of time between different side chain carbon atoms of Leu2 of Peptide-2 and Phe126 of MD2 (Figure 8), it can be deduced that they interact during the totality of the simulation, maintaining the Phe126 side chain inside the MD2, restricting its movement (with a backbone RMSD value approximately 3.5 Å, lower than the observed with the MD simulation of the TLR4/MD2 complex in absence of Peptide-2, Figures 8C,D). It has been reported that LPS and LPS-derived ligands that induce immersion of Phe126 in the MD2 pocket may result in an agonist effect, suggesting the role of this residue as a molecular switch for receptor activity (49, 71). Although it has also been reported that in the absence of ligand, the Phe126 side chain can find stabilization with Tyr131 of MD2 by π-π stacking interaction (72), the MD trajectories do not support the above, since the absence of a ligand in the simulation did not condition the position of Phe126 said chain. When the TLR4/MD2 complex was simulated by MD in the absence of Peptide-2, the Phe126 side chain underwent a high movement and maintained contact with the solvent, practically without interacting with MD2 residues, including Tyr131 side chain, remaining during the simulation in a position that suggests the non-activation of the receptor complex (Figure 8D). Even though the results of the MD simulations agree with the reports on the role played by Phe126 in the activation of the TLR4/MD2 receptor complex, it is not possible to fully understand or assert the effect of Peptide-2 based only on these observations.

Based on all the molecular docking and MD results, a Peptide-2 plausible binding mode exhibits the first eight residues at N-terminal surrounded by MD2 residues, and the remaining residues at C-terminal close to the TLR4 subunit. An inverted Peptide-2 orientation to the described is not plausible, due to the hydrophobic residues Leu2 and Leu6 of Peptide-2 would hardly find an optimal accommodation on TLR4, which does not present hydrophobic cavities to host these, as previously highlighted. Nonetheless, there is a need to obtain a TLR/MD2/Peptide-2 crystallized complex in order to corroborate the present results. As of today, a couple of TLR4 ligands beyond LPS have been described, including HSPs, high mobility box group 1, oxidized LDL, saturated fatty acids, fusion protein from respiratory syncytial virus, hyaluronic acid, β-defensin 2, and the envelope protein of mouse mammary tumor virus. The heterogeneous nature of all these molecules suggests that the TLR4-recognizing mechanisms are rather complex (73, 74).

As it has been previously mentioned, certain PAMPs and DAMPS may activate TLR4, among which HSP60 is a peculiar case, since it may play its part as both. Recognition of highly phylogenetically preserved epitopes in the protein leads to the onset of inflammatory responses and cell death (57), but also depending on HSP60 concentration and structural form (complete protein or peptides), an anti-inflammatory response could be exerted (18, 21, 75). The body of knowledge covering HSP60 and the particular role its peptides play in inducing pro- and anti-inflammatory processes as part of the pathophysiology of cardiovascular diseases is mostly derived from studying the cells of the immune system, setting the tone for immunomodulatory strategies aiming at halting the progression of tissue damage by tilting the inflammatory scale from a proinflammatory state toward an anti-inflammatory one. Having this goal in mind, one of the most explored options over the last years has been immunization using HSP60 peptides, seeking after the ideal candidates for the development of vaccines to provide novel therapies for the treatment of cardiovascular disease as a result of peripheral tolerance induction to the HSPs and its peptides (75). The aforementioned results point to the fact that the functional response with HSP60 peptides is dependent on the particular sequences utilized, and no “one size fits all” event should be expected. This urges research to test all potential antigens to vaccines using HSP60 peptides in different types of cells to assess their cytotoxic potential before exploring their therapeutic usefulness in more intricate disease models.

We initially explored the potential effect of a synthesized Peptide-2 in an *in vitro* model to explore its effects against LPS-induced cell viability reduction. A first approach into the possible applications of Peptide-2 was obtained by initial and exploratory assays (Supplementary Figure 5). Ventricular cardiomyoblasts (H9c2 cells) were utilized as a surrogate model from primary cardiomyocytes, where an important advantage related to their use is that they reduce, in exploratory stages of projects- like our case-, the need for animal studies and approximates and resembles to the following exploratory model (rat or mice). H9c2 represent a good study model since they share important features with primary cardiomyocytes, such as plasma membrane morphology, electrophysical properties, and the expression and signaling mechanisms of G proteins. We are aware that the final goal is to get closer to the mechanisms exerted in humans, the reason why human cell lines such as coronary artery smooth muscle cells (HCASMC), cardiomyocytes (HCM or AC16), cardiac fibroblast (HCF), microvascular endothelial cells (HCMEC), to mention some, must be used.

Nevertheless, H9c2 cardiomyoblasts also respond to neurohormones, such as Ang II and endothelin-1, which are potent stimuli capable of inducing hypertrophy, as demonstrated by a rearrangement of the cytoskeleton and expression of associated fetal genes in these cells (76, 77). Our initial results showed a significant reduction on cell viability exerted by LPS treatment, as an indicative of an LPS-induced TLR4 activation, and a significant viability increment by Peptide-2, in which it was clear that a positive effect on cell viability was obtained compared to the TLR4 agonist, LPS. Moreover, to determine Peptide-2 function as a possible TLR4 modulator, the competitive assay co-incubating LPS plus Peptide-2 showed that the peptide was able to prevent LPS-induced cell viability reduction, demonstrated by a substantial improvement in cell survival (Supplementary Figure 5).

Our *in silico* data open new insights on the understanding of HSP60/TLR4/MD2 interactions, even if we are not able to determine the specific mechanisms obtained in our preliminary *in vitro* assays exerted by Peptide-2. We should consider the molecular and supramolecular physical requirements of other HSP60-based peptide sequences for high-affinity and functional interactions with TLR4-MD-2, like LPS, and hence a prospective antagonist or agonist complete interpretation. As we know, it has been recognized that there are other mediators in the response to LPS besides MD-2 (also known as lymphocyte antigen 96), others such as CD14 and the LPS-binding protein (LBP) (78), so further exploration is necessary. Although we obtained the positive effects of Peptide-2 against LPS-induced cell viability, we are fully aware that further refining experiments must be performed in a second stage, using TLR4 specific antagonist, measuring cell viability by annexin V and propidium iodide staining, and determining proinflammatory and cell death, and other biochemical markers by protein expression to confirm that our observations are mediated by specific activation or inhibition of TLR4 pathway. On the other hand, an *in vivo* model must be conducted to consolidate our preliminary observations. To conclude, our results constitute a first theoretical approach to the binding mode of an HSP60-derived peptide with the TLR4/MD-2 complex in the absence of crystallographic structures. We propose the plausibility of this peptidic approach for TLR4 modulation as a promising therapy relevant to the treatment of TLR4-related cardiovascular diseases.

## Data Availability Statement

The raw data supporting the conclusions of this article will be made available by the authors, without undue reservation.

## Author Contributions

RV-C, JL-A, and CG-B performed the experiments, designed the studies, analyzed the data, and wrote the manuscript. All authors contributed to the article and approved the submitted version.

## Conflict of Interest

The authors declare that the research was conducted in the absence of any commercial or financial relationships that could be construed as a potential conflict of interest.

## Publisher’s Note

All claims expressed in this article are solely those of the authors and do not necessarily represent those of their affiliated organizations, or those of the publisher, the editors and the reviewers. Any product that may be evaluated in this article, or claim that may be made by its manufacturer, is not guaranteed or endorsed by the publisher.
