# Supplementary material for: HSP60-Derived Peptide as an LPS/TLR4 Modulator: An in silico Approach
Source: Front Cardiovasc Med. 2022 Apr 1;9:731376. doi: 10.3389/fcvm.2022.731376 (PMC9010565; doi:10.3389/fcvm.2022.731376)
Supplement: Supplementary file 1 [file Data_Sheet_1.PDF]

Supplementary Material

Table of content

List of Investigators ……………………………………………………………………1

Supplementary Methods ………………………………………………………………1

Supplementary Results …………………………………………………………...2

Supplementary Figures …………………………………………………………...3

Supplementary Tables …………………………………………………………....7

Supplementary Reference ………………………………………………………...7

1 List of investigators

Rafael Gustavo Vila-Casahonda[1†], Jorge Lozano-Aponte[2†], Carlos Enrique Guerrero-Beltrán[1*]
[1]Tecnologico de Monterrey, Medicina Cardiovascular y Metabolómica, Escuela de Medicina y Ciencias de la Salud, Tecnologico de Monterrey, Mexico
[2] Tecnologico de Monterrey, Escuela de Ingeniería y Ciencias, Tecnologico de Monterrey, Mexico
*Correspondence: Carlos Enrique Guerrero-Beltrán, Tecnologico de Monterrey, Escuela de Medicina y Ciencias de la Salud, Mexico. enriqueguerrero@tec.mx

2 Supplementary Methods

***In vitro* assays**

Cardiomyoblast cells H9c2 line (ATCC® CRL-1446) were cultured using DMEM (Dulbecco's Modified Eagle's Medium, Sigma, D7777) as culture medium, supplemented with 10% FBS (Fetal Bovine Serum, Biowest, S1650) and streptomycin (100mg / mL; GIBCO, 15140). Cells were incubated in 95% air- 5% CO_2 at 37 °C. Cell culture passages were performed when they reached 80% confluence.

To determine the effect of LPS (Sigma-Aldrich, USA) and HSP60 Peptide-2 (derived from *M. bovis*; RS Synthesis, LLC Louisville, KY) in rat cardiomyoblasts, H9c2 cells were cultured at densities of 1.75 x 103 in 200 µL of DMEM enriched with 10% FBS in 96-well plates. The following day, the medium was removed from the cells, washed with serum-free DMEM, and replaced by DMEM depleted in 1% FBS and its incubation was found for 24 h. Afterwards, the cells were treated with the different experimental conditions: control (incubated only with DMEM + SBF1% and an equivalent

volume of PBS to which the peptide that serve as treatments are prepared), LPS (100 µg/mL, as a positive control for TLR4 activation), and 3 concentrations of Peptide-2 (100, 200, and 400 µg/mL). At least three replicates of each experiment were generated for the purposes of statistical analysis of the results. To determine cell viability after the time of treatment with the experimental groups, cells were incubated with Alamar blue (Sigma-Aldrich, USA) (1:10) for 3 hours at 37 °C. This procedure measures the metabolic activity of cells through their ability to reduce resazurin (alamar blue) to resorufin (its fluorescent form) by oxide reductases that are mainly found in the mitochondria of living cells, which can be quantified by means of spectrophotometry at wavelengths of 560 and 590nm for excitation and emission, respectively.
To determine the effect of Peptide-2 as a possible modulator, over LPS-induced cell viability reduction, a coincubation assay was performed following the previous stated protocol, with different experimental conditions: control (incubated only with DMEM), LPS (100 µg/mL), Peptide-2 (100 or 200 µg/mL) and LPS + Peptide-2 (100 µg/mL + 100 or 200 µg/mL, respectively, as marked). At least three replicates of each experiment were generated for the purposes of statistical analysis of the results.

Statistical analyses
Cell viability assay: Data were analyzed by ANOVA followed by Bonferroni's multiple comparisons test using Graph Pad InStat (Graph Pad Software, San Diego, CA). Data were expressed as means ± SD. A p value of <0.05 was considered statistically significant.

3 Supplementary Results

In vitro studies

The effect of Peptide-2 and LPS on cardiomyoblast viability

In order to explore a potential effect of Peptide-2 and LPS on cardiomyoblast cell activity, *in vitro* assays were performed on H9c2 cells. As shown in (**Supplementary Figure 5**), LPS decreased cell viability in a dose-dependent manner (100 and 200 µg/mL), showing an LPS capacity to activate TLR4. Exposure to Peptide-2 induced a significant increase in cell viability by 20% and 30% (using 100, 200, and 400 µg/mL), as compared to the control group (CT) as shown in (**Supplementary Figure 5**).

Peptide-2 protects against LPS-induced cell death

The previous results allowed us to design a competitive assay, co-incubating LPS+Peptide-2 to determine Peptide-2 function as a possible TLR4 antagonist, showing that Peptide-2 (100 and 200 µg/mL) was able to prevent LPS-induced cell viability reduction. As shown in (**Supplementary Figure 5)**, LPS treatment significantly reduced cell viability to 77% of the control value. Peptide-2 treatment was able to induce an increase in cell viability, confirming our previous results. Finally, Peptide-2 was able to significantly prevent the LPS-induced cell death in a 24h co-incubation of LPS+Peptide-2, reaching viability values as those obtained in the control group (up to 98% and 92% as compared to LPS group (**Supplementary Figure 5**).

4 Supplementary Figures

Supplementary Figure 1. Sequence alignment for human and murine TLR4 and MD2. (**A**) TLR4 sequence alignment with human sequence as query and murine as subject. (**B**) MD2 sequence alignment with human sequence as query and murine as subject.

A)

Score	Expect	Method	Identities	Positives	Gaps
1087 bits(2812)	0.0	Compositional matrix adjust.	557/824(68%)	658/824(79%)	4/824(0%)

```
Query  1    MMSASRLAGTLIPAMAFLSCVRPESWEPCVEVVPNITYQCMELNFYKIPDNLPFSTKNLD  60
            MM    LA TLI A+ F SC+ P S  PC+EVVPNITYQCM+    K+PD++P STKN+D
Sbjct  1    MMPPWLLARTLIMAL-FFSCLTPGSLNPCIEVVPNITYQCMDQKLSKVPDDIPSSTKNID  59

Query  61   LSFNPLRHLGSYSFFSFPELQVLDLSRCEIQTIEDGAYQSLSHLSTLILTGNPIQSLALG  120
            LSFNPL+ L SYSF +F ELQ LDLSRCEI+TIED A+  L HLS LILTGNPIQS + G
Sbjct  60   LSFNPLKILKSYSFSNFSELQWLDLSRCEIETIEDKAWHGLHHLSNLILTGNPIQSFSPG  119

Query  121  AFSGLSSLQKLVAVETNLASLENFPIGHLKTLKELNVAHNLIQSFKLPEYFSNLTNLEHL  180
            +FSGL+SL+ LVAVET LASLE+FPIG L TLK+LNVAHN I S KLP YFSNLTNL H+
Sbjct  120  SFSGLTSLENLVAVETKLASLESFPIGQLITLKKLNVAHNFIHSCKLPAYFSNLTNLVHV  179

Query  181  DLSSNKIQSIYCTDLRVLHQMPLLNLSLDLSLNPMNFIQPGAFKEIRLHKLTLRNNFDSL  240
            DLS N IQ+I   DL+ L + P +NLSLD+SLNP++FIQ  AF+ I+LH+LTLR NF+S
Sbjct  180  DLSYNYIQTITVNDLQFLRENPQVNLSLDMSLNPIDFIQDQAFQGIKLHELTLRGNFNSS  239

Query  241  NVMKTCIQGLAGLEVHRLVLGEFRNEGNLEKFDKSALEGLCNLTIEEFRLAYLDYYLDDI  300
            N+MKTC+Q LAGL VHRL+LGEF++E NLE F+ S +EGLC++TI+EFRL Y + + DDI
Sbjct  240  NIMKTCLQNLAGLHVHRLILGEFKDERNLEIFEPSIMEGLCDVTIDEFRLTYTNDFSDDI  299

Query  301  IDLFNCLTNVSSFSLVSVTIERVKDFSYNFGWQHLELVNCKFGQFPTLKLKSLKRLTFTS  360
            +  F+CL NVS+ SL  V+I+ ++D   +F WQ L ++ C+  QFPTL L  LK LT T
Sbjct  300  VK-FHCLANVSAMSLAGVSIKYLEDVPKHFKWQSLSIIRCQLKQFPTLDLPFLKSLTLTM  358

Query  361  NKGGNAFSEVDLPSLEFLDLSRNGLSFKGCCSQSDFGTTSLKYLDLSFNGVITMSSNFLG  420
            NKG  +F +V LPSL +LDLSRN LSF GCCS SD GT SL++LDLSFNG I MS+NF+G
Sbjct  359  NKGSISFKKVALPSLSYLDLSRNALSFSGCCSYSDLGTNSLRHLDLSFNGAIIMSANFMG  418

Query  421  LEQLEHLDFQHSNLKQMSEFSVFLSLRNLIYLDISHTHTRVAFNGIFNGLSSLEVLKMAG  480
            LE+L+HLDFQHS LK+++EFS FLSL  L+YLDIS+T+T++ F+GIF GL+SL  LKMAG
Sbjct  419  LEELQHLDFQHSTLKRVTEFSAFLSLEKLLYLDISYTNTKIDFDGIFLGLTSLNTLKMAG  478

Query  481  NSFQENFLPDIFTELRNLTFLDLSQCQLEQLSPTAFNSLSSLQVLNMSHNNFFSLDTFPY  540
            NSF++N L ++F    NLTFLDLS+CQLEQ+S   F++L  LQ+LNMSHNN   LD+  Y
Sbjct  479  NSFKDNTLSNVFANTTNLTFLDLSKCQLEQISWGVFDTLHRLQLLNMSHNNLLFLDSSHY  538

Query  541  KCLNSLQVLDYSLNHIMTSKKQELQHFPSSLAFLNLTQNDFACTCEHQSFLQWIKDQRQL  600
              L SL  LD S N I TSK   LQHFP SLAF NLT N  AC CEHQ FLQW+K+Q+Q
Sbjct  539  NQLYSLSTLDCSFNRIETSKGI-LQHFPKSLAFFNLTNNSVACICEHQKFLQWVKEQKQF  597

Query  601  LVEVERMECATPSDKQGMPVLSL-NITCQMNKTIIGVSVLSVLVVSVVAVLVYKFYFHLM  659
            LV VE+M CATP +     VL   N TC M KTII VSV+SV+VVS VA L+Y FYFHL+
Sbjct  598  LVNVEQMTCATPVEMNTSLVLDFNNSTCYMYKTIISVSVVSVIVVSTVAFLIYHFYFHLI  657

Query  660  LLAGCIKYGRGENIYDAFVIYSSQDEDWVRNELVKNLEEGVPPFQLCLHYRDFIPGVAIA  719
            L+AGC KY RGE+IYDAFVIYSSQ+EDWVRNELVKNLEEGVP F LCLHYRDFIPGVAIA
Sbjct  658  LIAGCKKYSRGESIYDAFVIYSSQNEDWVRNELVKNLEEGVPRFHLCLHYRDFIPGVAIA  717

Query  720  ANIIHEGFHKSRKVIVVVSQHFIQSRWCIFEYEIAQTWQFLSSRAGIIFIVLQKVEKTLL  779
            ANII EGFHKSRKVIVVVS+HFIQSRWCIFEYEIAQTWQFLSSR+GIIFIVL+KVEK+LL
Sbjct  718  ANIIQEGFHKSRKVIVVVSRHFIQSRWCIFEYEIAQTWQFLSSRSGIIFIVLEKVEKSLL  777

Query  780  RQQVELYRLLSRNTYLEWEDSVLGRHIFWRRLRKALLDGKSWNP  823
            RQQVELYRLLSRNTYLEWED+ LGRHIFWRRL+ ALLDGK+ NP
Sbjct  778  RQQVELYRLLSRNTYLEWEDNPLGRHIFWRRLKNALLDGKASNP  821
```

B)

Score	Expect	Method	Identities	Positives	Gaps
225 bits(574)	1e-81	Compositional matrix adjust.	102/160(64%)	128/160(80%)	0/160(0%)

```
Query  1    MLPFLFFSTLFSSIFTEAQKQYWVCNSSDASISYTYCDKMQYPISINVNPCIELKRSKGL  60
            MLPF+ FSTL S I TE++KQ W CNSSDA ISY+YCD +++PISI+  PCI L+ + G
Sbjct  1    MLPFILFSTLLSPILTESEKQQWFCNSSDAIISYSYCDHLKFPISISSEPCIRLRGTNGF  60

Query  61   LHIFYIPRRDLKQLYFNLYITVNTMNLPKRKEVICRGSDDDYSFCRALKGETVNTTISFS  120
            +H+ +IPR +LK LYFNL+I+VN++ LPKRKEV+C G DDDYSFCRALKGETVNT+I FS
Sbjct  61   VHVEFIPRGNLKYLYFNLFISVNSIELPKRKEVLCHGHDDDYSFCRALKGETVNTSIPFS  120

Query  121  FKGIKFSKGKYKCVVEAISGSPEEMLFCLEFVILHQPNSN  160
            F+GI F KG Y+CV EAI+G  EE LFCL F I+H+ + N
Sbjct  121  FEGILFPKGHYRCVAEAIAGDTEEKLFCLNFTIIHRRDVN  160
```

Supplementary Figure 2. Comparative matrix of interactions between experimental Peptide-2, Eritoran and LPS with literature in (mTLR4/MD2)^2. (**A**) Comparative matrix for interactions obtained experimentally for ligands and reported in literature for MD2 subunit. Bold and underlined residues show matches between experimental and reported values. Red residues belong to Peptide-2 and are placed beneath the residue they interact with. (**B**) Comparative matrix for interactions obtained experimentally for ligands and reported in literature for TLR4 subunit. Bold and underlined residues show matches between experimental and reported values. Red residues belong to Peptide-2 and are placed beneath the residue they interact with. (**C**) Comparative matrix for interactions obtained experimentally for ligands and reported in literature for TLR4* subunit. Bold and underlined residues show matches between experimental and reported values. Red residues belong to Peptide-2 and are placed beneath the residue they interact with. EDM, EDH and EMDM refer to Experimental Docking Monomer, Experimental Docking Heterodimer and Experimental Molecular Dynamics Monomer respectively.

A)

MD2																			
Ligand	Reference	Interactions																	
LPS	Ohtho,U. et al.(2012)									Tyr 102	Pro 118	Phe 119	**Ser 120**	Phe 121	**Glu 122**	**Gly 123**	**Ile 124**		
	Wang,Y. et al. (2016)	Leu 78	Ile 80	Val 82	**Arg 90**									Phe 121	**Glu 122**	**Gly 123**		Leu 125	Phe 126
	EDM				**Arg 90**								**Ser 120**		**Glu 122**	**Gly 123**	**Ile 124**		
	EDH				**Arg 90**								**Ser 120**		**Glu 122**	**Gly 123**	**Ile 124**		
Peptide 2 (Monomeric)	EDM				Arg 90<br>Arg 11	Lys 91<br>Lys 14	Glu 92<br>Arg 11<br>Gly 15	Val 93<br>Met 10<br>Gly 15	His 96<br>Gly 15	Tyr 102<br>Met 10									
Peptide 2 (Monomeric)	EMDM				Arg 90<br>Gly 1<br>Glu 8		Glu 92<br>Gly 8<br>Arg 11	Val 93<br>Met 10					Ser 120<br>Leu 4			Gly 123<br>Gln 3			
Peptide 2 (Heterodimeric)	EDH				Arg 90<br>Gln 3		Glu 92<br>Gln 3			Tyr 102<br>Glu 8							Ile 124<br>Gly 1		
Eritoran	EDM				Arg 90		Glu 92	Val 93					Ser 120						
	EDH				Arg 90		Glu 92			Tyr 102	Pro 118		Ser 120						

B)

TLR4													
Ligand	Reference	Interactions											
LPS	Ohtho et al.(2012)	**Lys 263**	Lys 319	Arg 337				**Lys 360**					
	Wang, Y., Su et al	**Lys 263**											
	EDM	**Lys 263**						**Lys 360**					
	EDH	**Lys 263**						**Lys 360**					
Peptide 2 (Monomeric)	EDM						Asn 359<br>Asp 13		Gly 361<br>Asp 13	Arg 380<br>Asp 13	Asn 381<br>Asp 13	Ala 382<br>Asp 13	
Peptide 2 (Monomeric)	EMDM									Arg 380<br>Gly 15			Asn 407<br>Lys 14
Peptide 2 (Heterodimeric)	EDH	Lys 263<br>Thr 7		Arg 337<br>Glu 8	Gln 339<br>Thr 7	Met 358<br>Glu 8	Asn 359<br>Glu 9			Arg 380<br>Phe 12	Asn 381<br>Phe 12	Ala 382<br>Phe 12<br>Asp 13	
Eritoran	EDM							Lys 360					
	EDH	Not reported											

C)

TLR4 *						
Ligand	Reference	Interactions				
LPS	Ohtho,U. et al.(2012)	**Ser 413**			**Arg 434**	
	Wang,Y. et al. (2016)	**Ser 413**	Asn 415		**Arg 434**	Glu 437
	EDM	Not reported				
	EDH	**Ser 413**			**Arg 434**	
Peptide 2 (Monomeric)	EDM	Not reported				
Peptide 2 (Monomeric)	EMDM	Not reported				
Peptide 2 (Heterodimeric)	EDH			Lys 433<br>Asp 13	Arg 434<br>Gly 15<br>Arg 11<br>Asp 13	Glu 437<br>Gly 15
Eritoran	EDM	Not reported				
	EDH					Glu 437

Supplementary Figure 3. Root Mean Square Deviation and Radius of gyration of TLR4/MD2 backbone of the TLR4/MD2/Peptide-2 MD and TLR4/MD2 MD, both at 310 K for 100 ns. (A) Root mean square deviation or RMSD of TLR4/MD2 backbone (Å, Y axis) *vs.* Time (ns, X axis) plot, **(B)** Radius of gyration or Rg of TLR4/MD2 backbone (nm, Y axis) *vs.* Time (ns, X axis) plot. Light blue and yellow lines represent the average value between each simulation step. Average values are shown at the bottom of each graph. To calculate and plot the RMSD, the MD resulting conformations were previously aligned using the "Trajectory tool" of VMD package [38]. Vertical black and red lines are due periodic boundary effects. **(C)** Molecular dynamics trajectory superposition of TLR4/MD2 complex (TLR4/MD2 MD, in absence of Peptide-2). **(D)** Molecular dynamics trajectory superposition of TLR4/MD2 complex (TLR4/MD2/Peptide-2 MD, in presence of Peptide-2). **C** and **D** trajectories were aligned with VMD "Trajectory tool" and were tracked every 20 steps over a total of 10000, giving a total of 500 superposed conformations displayed without hydrogen atoms.

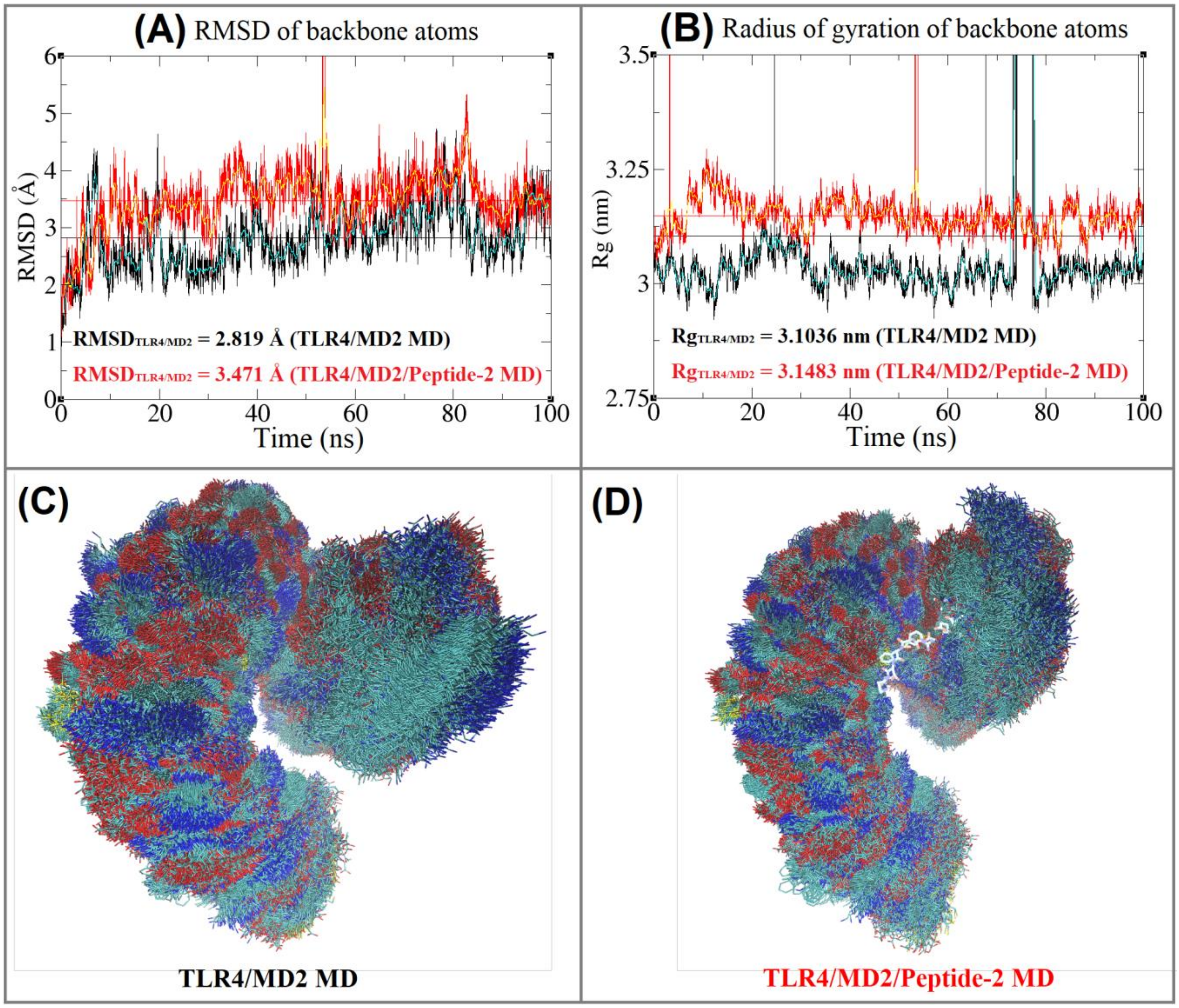

Supplementary Figure 4. **Autodock Vina Scoring Function.** The scoring function shows no clear description of methods other than *ftitj* term refers to the assigned score of an atom in terms of interactions, type, and interatomic distance (r_{ij}) and ht_it_j term is a weighted sum of steric interaction. It could be expected that resulting *in silico* values do not necessarily match experimental results.

$$c = \sum_{i<j} ft_i t_j (r_{ij})$$

$$ft_i t_j (r_{ij}) \equiv ht_i t_j (d_{ij})$$

Supplementary Figure 5. Peptide-2 protects against LPS-induced cell viability reduction on cardiomyoblast. (A) Treatment with LPS and Peptide-2 induced a reduction and increase in cell viability, respectively (n=5 per group). ***p<0.001 *vs.* control group (CT). Values are mean ±SEM. **(B)** Treatment with Peptide-2 prevented LPS-induced cell death (n=5 per group). ***p<0.0001 *vs.* control group; ## p<0.001 *vs.* LPS group (CT). Values are mean ±SEM.

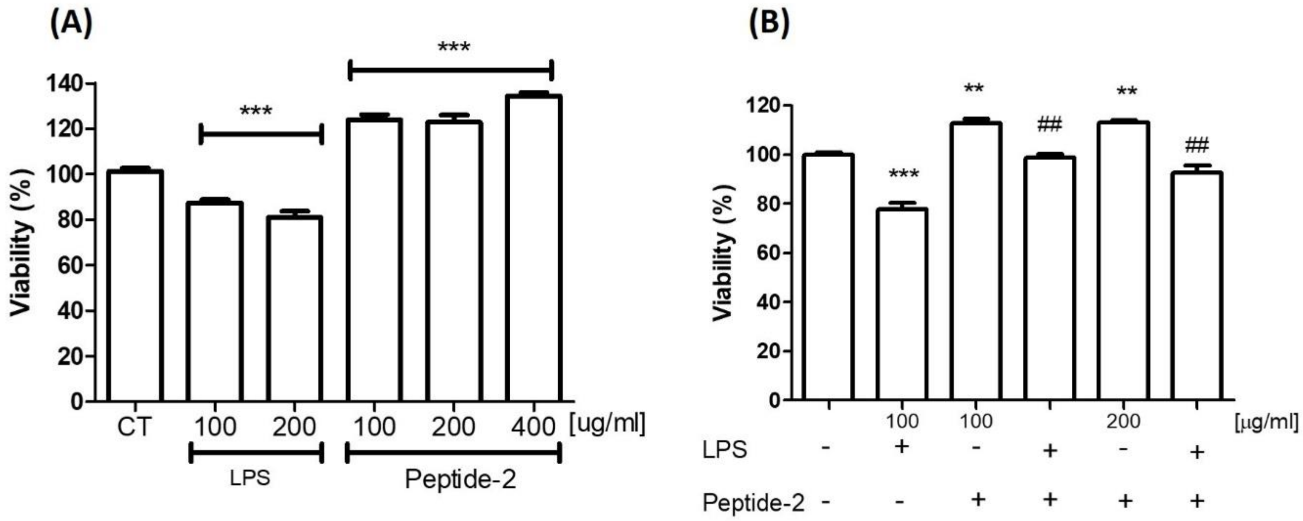

5 Supplementary Tables

Supplementary Table 1. Mean values of RMSD, Rg and 95% confidence intervals (CI95%) calculated for different molecular structures simulated by molecular dynamics at 310 K for 100 ns. On each case, CI95% was calculated by Z test with MiniTab 19 (1).

**Complex simulated by MD**	**RSMD of TLR4/MD2 backbone (Å)**	**RMSD - CI95% (Å)**	**Rg of TLR4/MD2 backbone (nm)**	**Rg - CI95% (nm)**
TLR4/MD2	2.819	2.808 - 2.829	3.1036	3.0958 - 3.1115
TLR4/MD2/Peptide-2	3.471	3.452 - 3.489	3.1483	3.1472 - 3.1498

Supplementary Table 2. Summary of interactions between TLR4/MD2 complex and Peptide-2 residues observed by molecular dynamics at 310 K. Single O or N: backbone residue atoms. NH1, NH2: nitrogen atoms at the end of the arginine side chain. NE: nitrogen epsilon atom of arginine side chain. NZ: nitrogen atom of lysine side chain. O1, O2: glycine 1 oxygen atoms at the C-terminus of Peptide-2. OE1, OE2: oxygen atoms of glutamic acid side chain.

<table>
<tr><td colspan="7">H-bond interacting residues</td></tr>
<tr><td>MD2</td><td>Arg90.NH1</td><td>Arg90.NH2</td><td>Glu92.OE1_OE2</td><td>Val93.O</td><td>Ser120.N</td><td>Gly123.N</td></tr>
<tr><td>Peptide-2</td><td>Gly1.O</td><td>Glu8.OE1</td><td>Gly9.N
Arg11.NH1
Arg11.NH2</td><td>Met10.N</td><td>Leu4.O</td><td>Gln3.OE1</td></tr>
<tr><td>TLR4</td><td colspan="3">Arg380.NE</td><td colspan="3">Asn407.O</td></tr>
<tr><td>Peptide-2</td><td colspan="3">Gly15.O1
Gly15.O2</td><td colspan="3">Lys14.NZ</td></tr>
<tr><td colspan="7">Hydrophobic interactions</td></tr>
<tr><td>MD2</td><td colspan="2">Ile80 Leu87
Val82 Leu125
<u>Phe126</u>*</td><td colspan="2">Ile44 Phe76
Val63 Phe104
Phe65 Phe147
Leu74 Leu149</td><td>TLR4</td><td>Arg337
Met358
Arg360</td></tr>
<tr><td>Peptide-2</td><td colspan="2">Leu2</td><td colspan="2">Leu6</td><td colspan="2">Phe12</td></tr>
</table>

6 Supplementary Reference

1. Arend DN. Minitab 19. Champaign,Illinois:U.S. Army Corps of Engineers Research Laboratory: Choises; 1993.
